# Foil

**Published:** 1862-06

**Authors:** 


### FOIL.

WE are now working Leslie's adhesive foil, and must say we have never found any work better. It is not harsh, and yet very adhesive. What we are now using is certainly not inferior to any adhesive foil we have ever used. It packs nicely and adheres well. We can hardly expect to find better foil than that now manufactured by Mr. Leslie. We do not mean or insinuate any disparagement to any other manufacturer, but are glad to record the fact that we have one as good as the best in our midst.

